# Supplementary material for: Awareness and Use of Canine Quality of Life Assessment Tools in UK Veterinary Practice
Source: Animals (Basel). 2023 Mar 20;13(6):1105. doi: 10.3390/ani13061105 (PMC10044674; doi:10.3390/ani13061105)
Supplement: Supplementary file 1 [file animals-13-01105-s001.zip › S1.pdf]

Quality of life assessment in dogs in veterinary practice

Formal quality of life assessment questionnaires/tools in dogs have been developed by researchers, pharmaceutical companies and within practices. A team of researchers from the University of Bristol are interested in the opinions of veterinarians and veterinary nurses on the quality of life of dogs as well as the use of formal and informal quality of life assessment in UK practice.

This survey should take less than 10 minutes to complete. Submissions are welcome from **veterinary surgeons and veterinary nurses** (including locums) who currently work with dogs in veterinary practice in the UK. There are no right or wrong answers. The first section is for you to tell us your thoughts on quality of life and about the use of assessment tools in practice. The second section is some brief information on you and your practice.

Completing and submitting this survey implies your consent for the information provided to be used in our study. However, the survey is anonymous, and all information given will be kept strictly confidential. If there are any questions which you do not wish to answer please leave them blank.

Part 1: Quality of life in dogs

1. It is difficult to define ‘quality of life’. What is your understanding of the term ‘quality of life’ in dogs?

2. We are curious about your opinion on measuring QoL with the help of questionnaires. Do you think Quality of life in dogs can be:

i. Yes Not yet No No opnion

b. Defined
c. Measured objectively
d. Validly measured (valid= measured accurately)
e. Reliably measured (reliable= measured consistently)
f. Used in the clinic
g. Used in research
yes, not yet, no, no opinion

3. In what way do you currently form an opinion on a dog's QoL during (outpatient) consultation? (never, sometimes, always)

Intuition

Clinical experience

In your consultation

From another veterinary professional's consultation

A paper questionnaire

A computerised questionnaire

Other

If other, please state……………………………………..

4. What, in your opinion, would be the ideal way to form an opinion of a dog's QoL? (never/sometimes/always)

Intuition

Clinical experience

In your consultation

From another veterinary professional's consultation

A paper questionnaire

A computerised questionnaire

Other

If other please state………………………….

5. Several questionnaires/tools to measure quality of life in dogs have been developed. Are you familiar with the existence of these questionnaires? Yes/no/not sure

6. We are interested in comparing the use of QoL scoring tools with the use of pain scoring tools. Please state if you are familiar with, use currently , or have used the following:

Familiar with use currently have used (plus why are you using it), (what are its limitations)

Canine Brief Pain Inventory

Canine Orthopaedic Index

Glasgow Composite Pain Score

Helsinki Chronic Pain Index

Liverpool Osteoarthritis in Dogs Scale

PDSA PetWise MOT

Zoetis PetDialog app

Constructed our own in house *Please say what for* __________________

Other QOL score *Please state*___________________________________

Other Pain Score *Please state*________________________________

7. In this next part we are curious about your opinion on the importance of Quality of Life questionnaires in the care of dogs

   How relevant do you find the use of QoL questionnaires for:

   Routine treatment

   Treatment of a dog with chronic disease

   Treatment of a dog with unexplainable complaints

   Clinical research

   Improvement in general health of a dog

   Certain choices in your treatment

   Veterinary practice

8. In my opinion:
   It is completely my own choice whether or not I use QoL questionnaires
   I have the skill and knowledge to use QoL questionnaires (NO, Yes)

9. Assuming that valid and reliable QoL will be available in the future what would you in general think about using these QoL questionnaires yourself? (not useful-very useful 5 point scale)

10. Please indicate what aspects would keep you from using QoL questionnaires in clinical practice

    Your own priorities are different
    Insufficient knowledge about QoL
    Inexperience with questionnaires
    Resistance from owners
    Insufficient information on questionnaires
    Availability of standardised questionnaires
    Availability of extra working space for assessment
    Extra time for assessment of questionnaire
    Training in administering the questionnaire
    Training in the interpretation of the questionnaires
    No assistance in administering the questionnaire
    Other reason *Please state*___________________________

Other reason *Please state*______________________________
Other reason *Please state*______________________________

11. . Who do you think should be primarily responsible for administering QoL questionnaires to owners of dogs?

12. Assuming that a valid and reliable Quality of life questionnaire (paper or computer) was available in dogs.

Would you want to use QoL questionnaires in your treatment?

Do you plan to use questionnaire in your treatment?

(certainly not, probably not, maybe, probably will, surely will)

13. We are curious about what circumstances you would find an assessment of quality of life in dogs useful. Can you comment on this?

Part 2: General information

14. What is your gender?
    Male/Female/other/prefer not to say

15. Which age category applies to you? *Select one*
    16-25/26-35/36-45/46-55/56-65/66+

16. How long have you been working in veterinary practice (including career breaks)? *Select one*
    0-5 Years/6-10 years/11-15 years/16 years +

17. Which type of veterinary practice best applies to you *Select one*
    100% small animal general practice
    Mixed practice (please indicate the balance you work e.g. 50:50)___________________________
    Referral hospital
    Other (*please state)*_________________________________

18. Does your current practice have one or more branch practices? *[Locums please refer to the practice you have most recently worked]*
    Yes/No

19. Are you a veterinary nurse or veterinary surgeon?

    Vet (will continue to, q20)

    Nurse (will continue to q23)

20. **For veterinary surgeons,** where did you graduate for your veterinary degree?
    Bristol
    Edinburgh
    Glasgow
    Cambridge
    London
    Liverpool
    Nottingham
    Other (*please state*)_________________________

21. **For veterinary surgeons** Which of the following best describes your current position? (*Please select one*)
    - Sole principal
    - Director
    - Equity partner
    - Salaried partner
    - Full time assistant
    - Part time assistant
    - Locum
    - Consultant
    - Other

22. **For veterinary surgeons** Do you have any of the following qualifications in addition to your veterinary degree? (*tick all that apply)*:
    - Batchelors degree (non-veterinary)
    - Business-related qualification
    - Certificate (RCVS)
    - Diploma (RCVS European or American college)
    - Fellowship (RCVS)
    - Masters degree (Veterinary)
    - Masters degree (non-veterinary)
    - Overseas qualification
    - PGCE or equivalent teaching qualification
    - PhD
    - Other

For veterinary surgeons -please continue to question 27

23. **For veterinary nurses**, which of the following best describes you? (select one)
    - Registered VN
    - Listed VN
    - Qualified VN (not listed/registered)
    - Student VN
    - Qualified VCA, ANA
    - Unqualified VN assistant
    - Other

24. **For veterinary nurses** Are you head nurse? Yes/No

25. **For veterinary nurses** Are you currently acting as a locum? Yes/No

26. **For veterinary nurses** do you have or are you working towards any further qualification(s)? *tick all that apply*:

    Bachelors (not VN
    Masters (VN related)
    Masters (non-VN related)
    PGCE or equivalent teaching qualification
    PhD
    RCVS DipAVN
    Veterinary nursing degree (pre-qualification)
    Veterinary nursing degree (post qualification)
    Other

27. What is the first part of the postcode of your main workplace (NB this is to get an idea of where the responses come from, not to track individual responses).

Many thanks for completing this survey. Please use this space for any comments on quality of life in dogs, quality of life assessment tools and/or this survey

If you would like more information on this survey, or if your practice would like to be involved in further investigations on quality of life tools in dogs, please contact Claire Roberts at: claire.e.roberts@bristol.ac.uk
